# Antagonistic interactions between filamentous heterotrophs and the cyanobacterium *Nostoc muscorum*

**DOI:** 10.1186/1756-0500-4-357

**Published:** 2011-09-13

**Authors:** Miroslav Svercel, Bianca Saladin, Sofia J van Moorsel, Sarah Wolf, Homayoun C Bagheri

**Affiliations:** 1 Institute of Evolutionary Biology and Environmental Studies, University of Zurich, Winterthurerstrasse 190, CH-8057, Switzerland

## Abstract

**Background:**

Little is known about interactions between filamentous heterotrophs and filamentous cyanobacteria. Here, interactions between the filamentous heterotrophic bacteria *Fibrella aestuarina *(strain BUZ 2) and *Fibrisoma limi *(BUZ 3) with an axenic strain of the autotrophic filamentous cyanobacterium *Nostoc muscorum *(SAG 25.82) were studied in mixed cultures under nutrient rich (carbon source present in medium) and poor (carbon source absent in medium) conditions.

**Findings:**

*F. aestuarina *BUZ 2 significantly reduced the cyanobacterial population whereas *F. limi *BUZ 3 did not. Physical contact between heterotrophs and autotroph was observed and the cyanobacterial cells showed some level of damage and lysis. Therefore, either contact lysis or entrapment with production of extracellular compounds in close vicinity of host cells could be considered as potential modes of action.

The supernatants from pure heterotrophic cultures did not have an effect on *Nostoc *cultures. However, supernatant from mixed cultures of BUZ 2 and *Nostoc *had a negative effect on cyanobacterial growth, indicating that the lytic compounds were only produced in the presence of *Nostoc*.

The growth and survival of tested heterotrophs was enhanced by the presence of *Nostoc *or its metabolites, suggesting that the heterotrophs could utilize the autotrophs and its products as a nutrient source. However, the autotroph could withstand and out-compete the heterotrophs under nutrient poor conditions.

**Conclusions:**

Our results suggest that the nutrients in cultivation media, which boost or reduce the number of heterotrophs, were the important factor influencing the outcome of the interplay between filamentous heterotrophs and autotrophs. For better understanding of these interactions, additional research is needed. In particular, it is necessary to elucidate the mode of action for lysis by heterotrophs, and the possible defense mechanisms of the autotrophs.

## Background

Cyanobacteria are photoautotrophic bacteria obtaining their carbon and energy by photosynthesis, while heterotrophic bacteria rely on organic compounds as their carbon and energy source. Within their natural aquatic environment, autotrophic cyanobacteria and heterotrophic bacteria live in a close relationship and can interact in various ways (synergistic, neutral, antagonistic). At present, little is known about such interactions, especially between filamentous cyanobacteria and filamentous heterotrophs. Few studies touching those relationships are available, and most focus on the biocontrol of harmful cyanobacterial blooms [1-4].

In 2009, two novel filamentous heterotrophic genera belonging to the family *Cytophagaceae *from the phylum *Bacteroidetes *were isolated from mud in tidal flats [5,6]. Not much is known about their "behavior" or ecological roles. Therefore, a model organism representing filamentous cyanobacteria was chosen to study their interplay in mixed cultures. Furthermore, we were interested in the following aspects: i) the effect of these microbes on autotrophs in nutrient rich (carbon source in medium) and nutrient poor environments (no carbon source in medium); ii) effect of heterotrophic extracellular metabolites on the growth and survival of cyanobacteria; iii) potential physical contact between heterotrophs and autotrophs; iv) the effect of cyanobacteria and their extracellular metabolites on the heterotrophs.

## Materials and methods

### Bacterial strains and cultivation

We used two novel heterotrophic filamentous bacteria isolated from a mud sample from tidal flats in Fedderwardersiel, on the North Sea coast of Germany. These were *Fibrella aestuarina *strain BUZ 2^T^[6] and *Fibrisoma limi *strain BUZ 3^T ^[5], which were separately cultivated in 30 mL of Ancylobacter-Spirosoma medium (SM, DSMZ 7) at 29°C on a shaker (120 rpm) for two (BUZ 2) or four days (BUZ 3). The autotrophic axenic cyanobacterium, *Nostoc muscorum *SAG 25.82 (identical strains ATCC 27893; PCC 7120) obtained from the SAG culture collection at University of Gottingen (Germany) was cultivated in 100 mL of BG11 medium [7] at room temperature under continuous light (800 lux) and shaking (120 rpm) for 21 days before starting the experiment.

### Experimental design

In the experimental set up, we tested the following treatments [see Additional file 1]: effect of SM broth on cyanobacterium, effect of heterotrophs *Fibrella aestuarina *BUZ 2^T ^or *Fibrisoma limi *BUZ 3^T ^on cyanobacterium in the mixture of SM and BG11 media, effect of washed heterotrophs (in BG11) on cyanobacterium, effect of heterotrophic supernatants on cyanobacterium, effect of BG11 medium on heterotrophs, effect of cyanobacterium on heterotrophs and effect of cyanobacterial supernatant on heterotrophs.

The mixed cultures and controls grown in preparation listed in the next section were cultivated in four 24 wells microtiter plates (TPP, Trasandingen, Switzerland) [see Additional file 1] at room temperature, exposed to continuous light (800 lux) under shaking (120 rpm) for 21 days.

### Preparation of bacterial cultures

Prior to the experiment, the cultures of heterotrophs and cyanobacterium were checked microscopically for purity using an Olympus BX 51 (Germany; 20× and 40× objectives) and by plating on SM agar. To achieve experimental densities of bacteria (2 × 10^6 ^cell mL^-1 ^for *Nostoc *and 10^7 ^cells mL^-1 ^for heterotrophs), 20 μL subsamples were taken from grown cultures and filament length was measured microscopically (4× objective for *Nostoc*, 20× objective for heterotrophs) using a Neubauer-improved counting chamber (Paul Marienfeld GmbH & Co, Germany) in four repetitions. To extrapolate cell numbers from the measured filament length, a conversion factor of the estimated mean cell lengths of 4.0 *μ*m, 6.7 *μ*m and 5.9 *μ*m for *Nostoc*, BUZ 2 and BUZ 3 respectively was used from a previous study [8]. Furthermore, to obtain the number of colony forming units (CFU) for the heterotrophs, 100 μL subsamples of the starting cultures were decimally diluted and plated on SM agar, incubated at 29°C and counted after 3 and 5 days (for BUZ 2 and BUZ 3), respectively.

### Bacterial supernatant, cell to cell contact observations and washing

In order to obtain the bacterial supernatant, the full grown individual or mixed bacterial cultures (*Nostoc *with BUZ 2 after two days cultivation) were centrifuged (4500 × *g *for 20 min) to produce cell pellets. To eliminate any cells remaining in the supernatant, the latter were passed through a 0.2 μm filter (Whatman, Dassel, Germany). The supernatant was checked microscopically and by plating on SM agar or BG11 agar (*Nostoc*) for the presence of bacterial cells.

To investigate the potential of cell to cell contact as a possible mode of action between heterotrophs and cyanobacterium, 20 μL subsamples from microtiter plate wells were microscopically checked (20× and 40× objectives).

For the test treatment with washed heterotrophs, the BUZ 2 and BUZ 3 cultures were centrifuged (as above) and the pellet rinsed twice with fresh BG11 medium to remove SM broth residuals. The final volume of BG11 medium was encompassed to reach wished density of heterotrophs.

### Measurement of cell density and CFU numbers

To measure the density of cyanobacteria, 20 μL subsamples were taken from three selected wells for each tested treatment in microtiter plates (containing cyanobacterium) on days 3, 7, 11 and 21 of cultivation and placed in a Neubauer-improved counting chamber. Two pictures per selected well were taken with a digital camera (Color View, Soft Imaging System) connected to a phase-contrast microscope (4× objective). The length of the filaments was determined using "Soft Imaging System CellF" from Olympus (Germany). Cell numbers of the autotroph were calculated from measured filament lengths as mentioned previously.

To count the CFU numbers of the two heterotrophs, 100 μL subsamples from two selected wells in each column (treatments containing BUZ 2 or BUZ 3) were decimally diluted and plated on SM agar on days 3, 7, 11 and 21 of cultivation. The CFU were counted after 3 or 5 days (for BUZ 3) of cultivation at 29°C in the dark.

### Algicidal effect

The possible percent algicidal effect of heterotrophs on cyanobacterium was calculated according Jung et al. [9] as: **(1 - T/C) × 100**, where T is the cyanobacterial density in the presence of heterotroph (treatment group), C is the cyanobacterial density in the control (two control groups in absence of the heterotrophs: i) control in BG11, ii) control in a mixture of SM and BG11).

### Statistics

Cell numbers were log-transformed for statistical analyses. Analysis of variance was performed at the significance level of *P *< 0.05, using SPSS (version 17.0, SPSS Inc. Chicago, IL) and means were separated using Tukey's test (*P *< 0.05). The whole experiment with experimental set up was done twice and representative data are shown.

## Results

### Effect of media on autotroph

**Figure 1:**
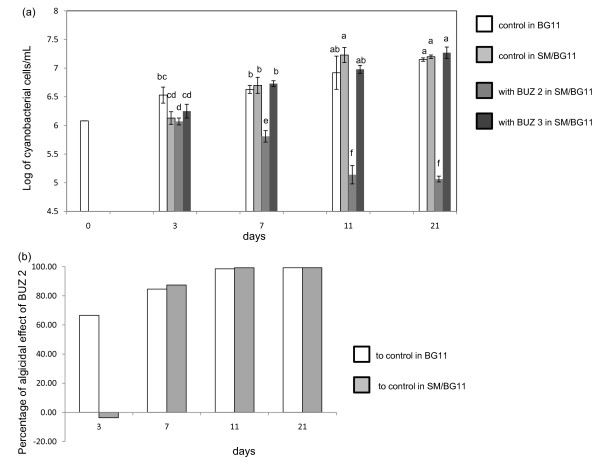
**Effect of media and heterotrophs on autotrophs and algicidal effect**. Effect of media and heterotrophs on autotrophs, and algicidal effect of BUZ 2 on autotrophs; (a) White bars: autotroph in BG11 (white bar at day 0 is common starting cell density for all treatments), light gray bars: autotroph in the mixture of SM/BG11 (1:1), dark gray bars: autotroph in the presence of heterotrophic BUZ 2, black bars: autotroph in the presence of heterotrophic BUZ 3 in SM/BG11. Values represent the means from four to eight cell counts per treatment ± st. dev. Significant differences are indicated by different letter according to a *P *value of 0.05. (b) Algicidal effect of BUZ 2 on the autotroph calculated according to Jung et al (2008); white bars: algicidal effect compared to cyanobacterial culture in BG11, grey bars: algicidal effect compared to cyanobacterial culture in SM/BG11 mixture (1:1).

In the absence of heterotrophic bacteria, the numbers of cyanobacterial cells increased during the cultivation period. The mixture of SM/BG11 media (1:1) did not negatively influence the cyanobacterial growth during the experiment (Figure 1a). There were slightly less cyanobacteria in mixture of SM/BG11 media than in only BG11. However, this was observed only in the first measurement (after 3 days) (Figure 1a). When *Nostoc *was cultivated in a SM/BG11 mixed medium, there was a lower incidence of long filaments than in BG11 only. However, the difference between the mean lengths between these two treatments was not statistically significant (data not shown).

### Effect of heterotrophs on autotroph

**Figure 2:**
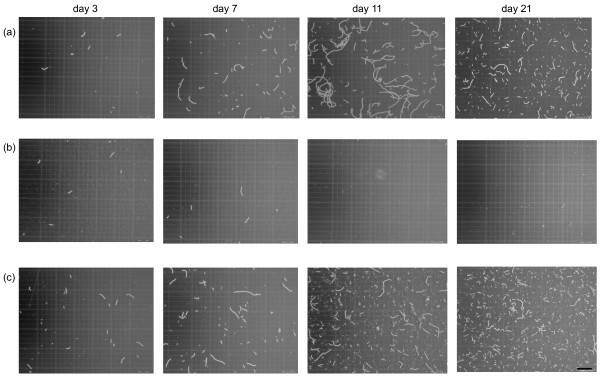
**Cyanobacteria in counting chambers in three different treatments**. Micrographs showing cyanobacterial filaments in three different treatments in counting chambers during the experimental period: (a) pure culture (control) in the mixture of SM/BG11, (b) in the presence of BUZ 2 in SM/BG11, and (c) in the presence of BUZ 3 in SM/BG11. Bar: 200 μm.

The strain BUZ 2 showed a strong negative effect on the autotroph when the cultivation medium was composed of SM/BG11. No cyanobacterial filaments were observed (only single remaining cells) after the second measurement (7 d) (Figure 1a and 2b). The calculated algicidal effect reached 98% after the second measurement (7d) and subsequently increased to nearly 100% (Figure 1b). In the absence of SM medium, the washed BUZ 2 culture was not able to diminish the cyanobacterial population. Unexpectedly in this case, *Nostoc *continued growing slowly (data not shown).

Strain BUZ 3 had a slight positive effect on the number of cyanobacterial cells (Figure 1a). However, the average length of *Nostoc *filaments was shorter when cultivated in the presence of heterotrophic BUZ 3 in SM/BG11 cocktail. It is inconclusive whether this was caused by BUZ 3, SM medium, or both. The washed cultures of BUZ 3 had no significant effect on numbers of cyanobacterial cells or filament length (data not shown).

### Mode of action

**Figure 3:**
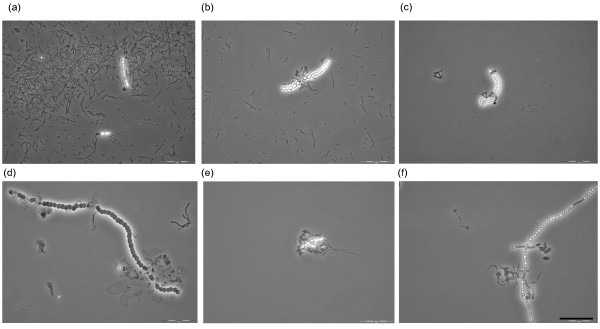
**Interactions between filamentous heterotrophs and autotrophs**. Micrographs illustrating potential interactions between filamentous heterotrophs (black-gray filaments) and autotrophs (bigger bright cell-chains or cells). (a, b) BUZ 2 connected to *Nostoc *filaments or remaining cells in SM/BG11, (c) BUZ 3 free in SM/BG11 medium and connected to cyanobacterium (in the middle), (d) BUZ 2 in washed culture (without SM) breaking the *Nostoc *filament, (e) Bundle of BUZ 2 (washed) filaments covering and attaching to the autotroph, (f) Aggregate of BUZ 3 (washed) filaments covering short *Nostoc *filaments. Bar: 50 μm.

Physical interactions and contacts between the tested filamentous heterotrophs and autotroph were observed on the microscopic pictures, and the cyanobacterial cells showed some level of damage and lysis (Figure 3). Such cell to cell contacts were found in the washed cultures of heterotrophs as well. The presence of heterotrophic supernatants from axenic cultures did not affect *Nostoc *numbers negatively. The results were similar as in control treatments. Therefore, we assumed that no lytic compounds were produced in the pure heterotrophic cultures prior the experiment. However, the supernatant extracted from mixed, fully grown cultures of *Nostoc *and BUZ 2 showed significant negative effects on the number of cyanobacterial cells. After 3 days, the algicidal effect reached 62%, and after the second and third measurements it had reached above 91%. In addition, the length and numbers of multicellular cyanobacterial filaments significantly decreased (data not shown). This means that BUZ 2 is producing lytic compound only in the presence of cyanobacterium.

### Effect of media and autotroph on heterotrophs

**Figure 4:**
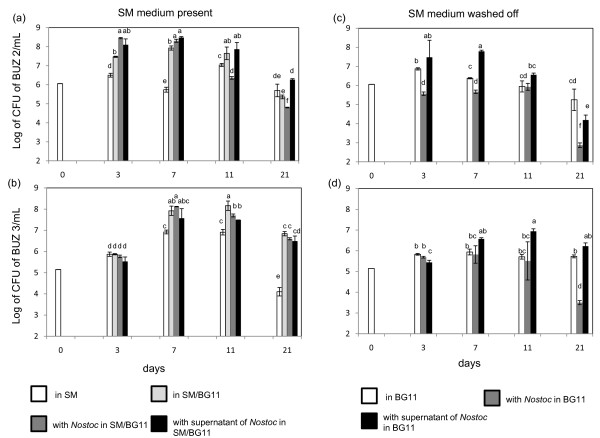
**Effect of media and autotroph on the heterotrophs**. Effect of media and autotroph on the survival of heterotrophs BUZ 2 and BUZ 3: (a, b) in the presence of SM; white bars: heterotrophs in SM (white bar at day 0 is common starting CFU for all treatments), light gray bars: heterotrophs in the mixture of SM/BG11 media (1:1), dark gray bars: heterotrophs in the mixture of SM and cyanobacterial culture in BG11, black bars: heterotrophs in the mixture of SM and supernatant of cyanobacterial culture in BG11; (c, d) in the absence of SM (washed cultures with BG11), white bars: heterotrophs in BG11 (white bar at day 0 is common starting CFU for all treatments), grey bars: in the presence of cyanobacterial culture, black bars: in the mixture of BG11 with cyanobacterial supernatant. Values represent the means from four to six CFU counts per treatment ± st. dev. Within each panel, significant differences are indicated by different letter according to a *P *value of 0.05.

Growth of *Fibrella aestuarina *(BUZ 2) was stimulated significantly in the presence of cyanobacterium when cultivated in the mixture of SM/BG11 media (there was already an effect after the first measurement on the third day; Figure 4a). However, the CFU numbers decreased rapidly by the third and the fourth measurements (11 and 21 days). This is probably caused by depletion of nutrients in the medium. BUZ 2 grew better in treatments that included BG11 in comparison to cultures with only SM (Figure 4a). However, when grown in only BG11 after washing (Figure 4c), BUZ 2 did not grow significantly better than in SM. Interestingly, when cultivated in the presence of *Nostoc*, there was a significant decreased of CFU numbers in comparison to treatment with BG11 by the end of the experiment after 21 days (Figure 4c). When cyanobacterial extract was added to washed BUZ 2, higher CFUs were detected in comparison to treatments with BG11 and BG11/cyanobacterium in three of four measurements (Figure 4c). This means that BUZ 2 was able to grow on cyanobacterial metabolites.

Similar to BUZ 2, *Fibrisoma limi *(BUZ 3) was influenced positively when grown in the following three mixtures: i) SM/*Noctoc *in BG11 (Figure 4b), ii) SM/BG11 and iii) SM/cyanobacterial extract in BG11. However, no significant differences were found in the first measurement (after 3 days), which could be due to the generally slower growth of BUZ 3 in comparison to BUZ 2 (Filippini, personal communication). Cyanobacterial extract boosted the growth of washed BUZ 3 in the second and third measurement. The CFU numbers of washed heterotroph in BG11 did not change significantly within the cultivation period. However, CFUs were significantly lower in the last measurement (21 d) when washed BUZ 3 was cultivated with *Nostoc *(Figure 4d).

## Discussion

### Effect of media and heterotrophs on autotroph

Many phylogenetically diverse heterotrophic bacteria have been isolated and identified from environments dominated by cyanobacteria (e.g, cyanobacterial blooms) [1]. In mixed cultures, the interaction between cyanobacteria and other bacteria can vary from mutualistic (mostly connected with nutrient exchanges and cycling) [10] to growth-inhibition or cell-lysis of one of the actors.

Cultivation conditions are a very important issue affecting the outcome of these interactions. From our preliminary experiment, SM/BG11 broth showed the least adverse influence on *Nostoc *growth from the various broths tested (including media with carbon sources, data not shown). However, when adding SM to cyanobacteria, their growth was negatively affected slightly for a few initial days due to the new condition. But later, as shown by the second measurement (day 7), the *Nostoc *cultures appeared to adapt to this cocktail and were growing even better than in pure BG11 (Figure 1a). Indeed, according the literature, *Nostoc muscorum *can grow heterotrophically if glucose is present [11]; as is the case in SM medium.

BUZ 2 had a strong significant negative effect on cyanobacterial growth under nutrient rich conditions (SM/BG11). However it had no effect under nutrient poor conditions (SM washed out with BG11). One explanation could be that BUZ 2 first fed on SM and used trace elements, iron and nitrate from BG11, reached high cell numbers, and after finishing the carbon sources, started to attack the cyanobacterial filaments. Hence the interaction could start as nutrient and space competition and later switch to predation. In washed cultures (nutrient poor), *Fibrella *was not able to reach a density of cells which would have allowed them to destroy cyanobacterial filaments. Indeed, the ratio between pathogen (or predator) and host (or prey) is an important factor in natural pathogenicity (or predation) and also species dependent [12]. The ratios of BUZ 2 to *Nostoc *cells were very different in SM/BG11 in comparison to washed cultures in BG11 media. In SM/BG11, the cell ratios progressed from 1:1 (starting point), to 241:1 (3 d), 314:1 (6 d), 17:1 (11 d) and 1:2 (21 d). Conversely, in nutrient poor BG11 medium, the ratios favoured *Nostoc *with values 1:1, 1:7, 1:12 and 1:18218. Therefore, by varying the ratio in favor of BUZ 2, it could be still possible that the heterotroph may harm the cyanobacterium under nutrient poor conditions, but this remains to be tested.

*Fibrisoma sp*. strain BUZ 3 had no significant effect on the autotroph. Microscopic pictures (Figure 3 c and f) suggest physical contact between BUZ 3 and cyanobacterial cells, but cell numbers of BUZ 3 might have been too low to significantly harm cyanobacterial growth. Indeed, the ratios of BUZ 3 to *Nostoc *(maximal ratio was 24:1 after seven days) did not reach as high as in the BUZ 2 case. This might be due to their low growth rate (in comparison to BUZ 2) or to their physiological characteristics. In washed cultures in BG11, all of the ratios were tilted towards higher numbers of cyanobacteria (lowest cell ratio 1:3120 in the last measurement).

Negative effects of heterotrophic bacteria on cyanobacterial growth could be caused via different modes of action, including nutrient and space competition, production of secondary metabolites in the close vicinity of entrapped cyanobacteria (so called indirect way) and/or contact lysis and parasitism (direct way). The observed physical interaction between the filaments of the heterotrophs and the autotroph possibly imply either contact lysis or entrapment with production of extracellular compounds. This is in agreement with present literature, where the five members of the order *Sphingobacteriales *showed similar mechanisms when tested on filamentous or non-filamentous cyanobacteria [2-4]. Interestingly, extracts of both our tested heterotrophs had no effect on cyanobacteria. However, it should be noted that we had filtered the extract from heterotrophs that had been grown in the absence of cyanobacteria (in pure cultures). The possibility remained that production of harmful substances may depend on the presence of the prey (in our case cyanobacteria), or could be released only on the cell surface of the prey. This possibility was tested using the supernatant from mixed cultures of BUZ 2 and *Nostoc *cultivated together for two days. This supernatant displayed negative effects on cyanobacterial growth after three days. In comparison, Sallal [3] showed that *Flexibacter flexilis *adsorbed to *Oscillatoria williamsii *and then released a lysozyme lysing the cyanobacterium. Furthermore, extracellular metabolites in the cell filtrates (from pure bacterial cultures) led to growth inhibition. Rashidan and Bird [2] analyzed the mode of action of two *Cytophaga *strains on cyanobacteria. Given the fact that lysing enzymes were not secreted into the medium (extracts gained from pure and mixed cultures as well), and it appeared that no special attachment organelles exist on the surface of *Cytophaga*, it seems likely that surface lytic enzymes were involved in the lytic action [2]. The lytic bacteria also inhibited photosynthesis, glycolate dehydrogenase and nitrogenase. The wide host range of tested *Flexibacter *(which includes *Nostoc muscorum*) indicates the possibility of using those bacteria in controlling cyanobacterial growth [3].

A novel strain of filamentous helical *Saprospira *did not produced cyanobactericidal compounds into medium but showed group behaviour when cultivated together with filamentous *Anabaena *sp [4]. It formed three-dimensional reticular structures and lysed the host through direct contact. Furthermore, its group behavior greatly accelerated the cyanobactericidal process in comparison to individual filaments. Therefore a relevant question arises: do filamentous heterotrophs mainly act as a bundle of filaments or as single filaments? Unfortunately, only Shi et al. [4] explicitly mentioned that the *Saprospira *strain is filamentous and that it builds up such group structures. Figure 3e is an example indicating that the tested heterotrophs could aggregate and cover parts of cyanobacterial filaments. It is not known how often this happens, and whether this kind of behavior is representative for the tested heterotrophs. Further experiments could shed light on the occurrence and mechanism of this mode of action. Concerning other closely related taxa, *Flexibacter flexilis*, *Cytophaga *sp. (according Reichenbach [13]) and *Chytinophaga sancti *[14] should be filamentous. However, this could not be seen it the pictures of strains tested by Sallal [3] nor by Rashidan and Bird [2]. Therefore, additional studies are necessary. The appropriate use of electron microscopy and atomic force microscopy, as done by Malfatti and Azam [15] could clarify this issue.

### Effects of media and phototroph on heterotrophs

Our experimental set up allowed us to check the densities and survival of heterotrophs in different situations [see Additional file 1]. This issue is often not reported in lytic studies of cyanobacteria. We detected better growth of both heterotrophs in the mixture of SM/BG11 than in SM or BG11 alone (Figure 4). This could be expected as BG11 contains nitrate, iron and trace elements such as B, Mo, Co, Cu and Mn which are very important for heterotrophic metabolism. Furthermore, our tested heterotrophs could use cyanobacterial cells and cyanobacterial supernatant as nutrient sources (Figure 4). However, in the presence of *Nostoc*, the outcome of competition can depend on ratios between heterotroph and autotroph. Both heterotrophs could survive well when washed in BG11 (nutrient poor) during the whole experiment (Figure 4c, d). This was not always true when *Nostoc *was present. It is possible that *Nostoc *uses some strategy to out-compete the heterotrophs. An example are some strains of *Nostoc muscorum *that produce toxins which kill heterotrophs [16]. However, it is not known whether the strain we use produces toxins.

Our results suggest that the outcome of interaction between filamentous heterotroph and autotroph depends on the presence of nutrient in cultivation media, which boost or reduce the numbers of heterotrophs and hence change the ratios between the actors. To understand these interactions, additional research is needed. In particular, it is necessary to shed light unto the mode of action for lysis by heterotrophs and the possible defence mechanisms of the autotrophs.

## Competing interests

The authors declare that they have no competing interests.

## Authors' contributions

MS designed, performed the experiments and wrote the manuscript. BS, SJM and SW performed the experiments and helped with data and statistical analyses. HCB evaluated results and wrote the manuscript.

## Supplementary Material

Additional file 1 **Experimental set up**. Experimental set up in four 24-wells microtiter plates. The letters ABCD are repetitions of the same treatment in one column. Click here for file
