# Supplementary material for: Antagonistic interactions between filamentous heterotrophs and the cyanobacterium Nostoc muscorum
Source: BMC Res Notes. 2011 Sep 13;4:357. doi: 10.1186/1756-0500-4-357 (PMC3180475; doi:10.1186/1756-0500-4-357)
Supplement: Additional file 1 — Experimental set up. Experimental set up in four 24-wells microtiter plates. The letters ABCD are repetitions of the same treatment in one column. [file 1756-0500-4-357-S1.PPT]

## Slide 1
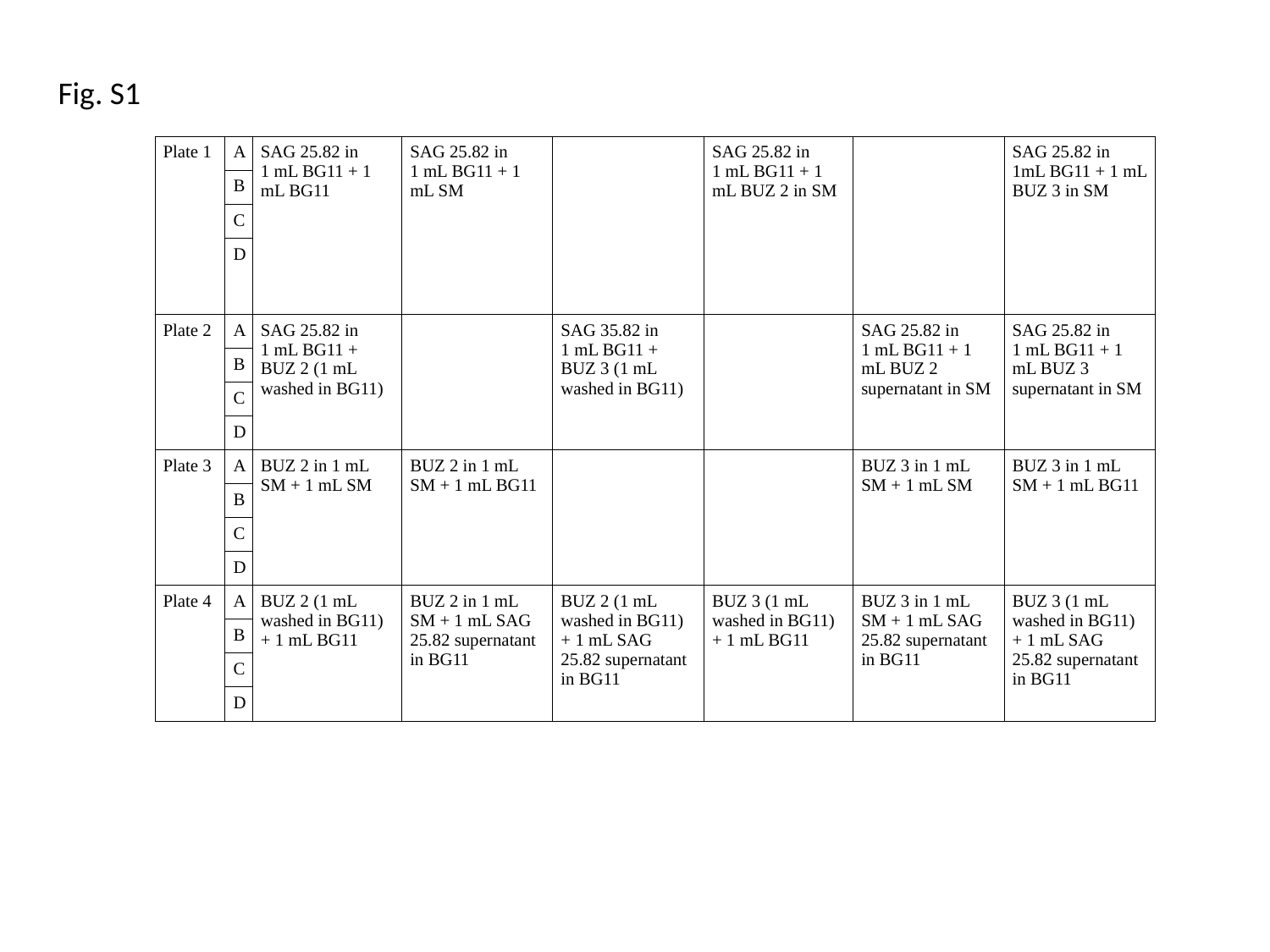

Fig. S1
| Plate 1 | A | SAG 25.82 in 1 mL BG11 + 1 mL BG11 | SAG 25.82 in 1 mL BG11 + 1 mL SM | | SAG 25.82 in 1 mL BG11 + 1 mL BUZ 2 in SM | | SAG 25.82 in 1mL BG11 + 1 mL BUZ 3 in SM |
| --- | --- | --- | --- | --- | --- | --- | --- |
| | B | | | | | | |
| | C | | | | | | |
| | D | | | | | | |
| Plate 2 | A | SAG 25.82 in 1 mL BG11 + BUZ 2 (1 mL washed in BG11) | | SAG 35.82 in 1 mL BG11 + BUZ 3 (1 mL washed in BG11) | | SAG 25.82 in 1 mL BG11 + 1 mL BUZ 2 supernatant in SM | SAG 25.82 in 1 mL BG11 + 1 mL BUZ 3 supernatant in SM |
| | B | | | | | | |
| | C | | | | | | |
| | D | | | | | | |
| Plate 3 | A | BUZ 2 in 1 mL SM + 1 mL SM | BUZ 2 in 1 mL SM + 1 mL BG11 | | | BUZ 3 in 1 mL SM + 1 mL SM | BUZ 3 in 1 mL SM + 1 mL BG11 |
| | B | | | | | | |
| | C | | | | | | |
| | D | | | | | | |
| Plate 4 | A | BUZ 2 (1 mL washed in BG11) + 1 mL BG11 | BUZ 2 in 1 mL SM + 1 mL SAG 25.82 supernatant in BG11 | BUZ 2 (1 mL washed in BG11) + 1 mL SAG 25.82 supernatant in BG11 | BUZ 3 (1 mL washed in BG11) + 1 mL BG11 | BUZ 3 in 1 mL SM + 1 mL SAG 25.82 supernatant in BG11 | BUZ 3 (1 mL washed in BG11) + 1 mL SAG 25.82 supernatant in BG11 |
| | B | | | | | | |
| | C | | | | | | |
| | D | | | | | | |
